# Living alone for people on dementia medication: related use of drugs

**DOI:** 10.18632/aging.104125

**Published:** 2020-10-21

**Authors:** Evi Zafeiridi, Alan J. McMichael, A. Peter Passmore, Bernadette McGuinness

**Affiliations:** 1 Centre for Public Health, Queen’s University Belfast, Belfast, United Kingdom

**Keywords:** dementia, living arrangements, medication, treatment, caregiver

## Abstract

Approximately one-third of people with dementia in the United Kingdom live alone. People living alone with dementia may receive different treatment for dementia and may have different comorbidities compared to people who live with a caregiver. This study explored differences in medication and demographic characteristics between people living alone with dementia and those living with a caregiver in Northern Ireland. People with dementia were identified through the first date that a dementia management medication was prescribed between 2010 and 2016. In total, 25,418 people were prescribed a dementia management medication. Data for whether people with dementia lived alone was extracted through the National Health Application and Infrastructure Services and from national datasets through the Honest Broker Service. Approximately 35% (n= 8,828) of people with dementia in Northern Ireland lived alone. People with dementia who lived alone were younger (mean= 75 years, SD= 8.50) compared to people who lived with a caregiver (mean= 77 years, SD= 7.82). Binary logistic regression highlighted that people who lived alone were more likely to be treated with donepezil medication for dementia and less likely to receive antidepressants. These findings indicate that living alone did not affect treatment for dementia and comorbidity medication in people on dementia medication.

## INTRODUCTION

Approximately one-third of community-dwelling people with dementia in the United Kingdom and the United States live alone; these rates, however, are higher in other countries, such as Germany (51%) and Sweden (46%) [1, 2]. In 2010, there were 141,460 people with dementia living alone in the United Kingdom [3].

The presence of a caregiver is likely to affect therapeutic decisions as informal primary caregivers can help ensure that the person with dementia receives treatment, help with drug adherence and help with the management of side effects of treatments [2]. Conversely, people with dementia living without a caregiver use health services at home more often than those living with other people [4, 5] and they are at an increased risk of hospitalisation [6] and institutionalisation [5–7].

One study exploring differences between people with dementia living alone with those who live with an informal caregiver found no differences in the management of medication-related problems, such as adverse reactions, as well as in nutrition, quality of life, or cognitive and functional impairment [1]. Other studies have shown that living alone with Alzheimer’s disease was associated with increased likelihood of receiving antidepressants, antipsychotics, hypnotics and sedative drugs in comparison to people who lived with an informal caregiver [2]. On the other hand, people living alone with dementia received cholinesterase inhibitors, cardiovascular medication and memantine less often [2].

Although past research has explored the unmet needs for people with dementia who live alone [3], including help with looking after their home, help with self-care, and having company; to the best of our knowledge, research on whether drug prescriptions and comorbidities differ for people who live alone is limited to findings from Sweden [2, 8] and France [6]. Therefore, this study investigated whether people with dementia who live alone in Northern Ireland receive the same treatment, as measured through dementia and comorbidity medications, with people who live with another person.

## RESULTS

### Differences in demographic characteristics

From 25,418 people with dementia (mean age 77.30 years, 65% females), 8,828 people lived alone and 12,200 people lived with another person in January 2010 (Table 1). The remaining 4,390 people had transitioned to a care home and were excluded from analysis. Compared to people living with another person, people living alone were significantly younger, lived in more deprived areas, were significantly more likely to live in rural areas (31%), were more likely to be females (65%) and were less likely to be married (40%) (Table 1). Donepezil was prescribed to people who lived alone more often compared with people who lived with a caregiver (31%). People living alone with dementia were prescribed anxiolytics less often (20%), as well as antidepressants (16%), hypnotics (19%), and anti-hypertensives (4%).

**Table 1:** Demographic characteristics.

	**Living alone**	**Living with another person**	**P-value**
**N (%)**	8,828 (34.73)	12,200 (48%)	
**Age, mean (SD)**	75.49 (8.50)	77.15 (7.82)	**<0.001^a^**
**Gender, n (%)**			**0.014^b^**
Males	3,144 (35.6)	4,546 (37.3)	
Females	5,684 (64.5)	7,654 (62.7)	
**Deprivation, mean (SD)**	5.42 (2.91)	5.76 (2.92)	**<0.001^a^**
**Area of living, n (%)**			**<0.001^b^**
Urban	5,872 (66.5)	8,395 (68.8)	
Rural	2,723 (30.9)	3,550 (29.1)	
**Marital status, n (%)**			**<0.001^b^**
Married	3,488 (39.5)	5,587 (45.8)	
Single	698 (7.9)	804 (6.6)	
Divorced	285 (3.2)	203 (1.7)	
Widowed	2,272 (25.7)	2,848 (23.3)	
**Dementia medication, n (%)**			
Memantine	2,558 (29.0)	3,614 (29.6)	0.309^b^
Donepezil	2,741 (31.0)	3,498 (28.7)	**<0.001^b^**
Galantamine	365 (4.1)	519 (4.3)	0.670^b^
Rivastigmine	347 (3.9)	506 (4.1)	0.431^b^
AChEI and memantine*	2,548 (28.9)	3,701 (30.3)	**<0.001^b^**
**Comorbidity medication, n (%)**			
Hypercholesterolemia	630 (7.1)	952 (7.8)	0.070^b^
Diabetes	224 (2.5)	363 (3.0)	0.057^b^
Hypertension	366 (4.1)	590 (4.8)	**0.018^b^**
Chronic anticoagulation	569 (6.4)	855 (7.0)	0.109^b^
Anxiolytics	1,798 (20.4)	2,746 (22.5)	**<0.001^b^**
Antidepressants	1,371 (15.5)	2,161 (17.7)	**<0.001^b^**
Parkinson’s disease	178 (2.0)	238 (2.0)	0.736^b^
Thyroid hormone problems	271 (3.1)	351 (2.9)	0.416^b^
Hypnotics	1,663 (18.8)	2,481 (20.3)	**0.007^b^**
Antipsychotics	1,776 (20.1)	2,638 (21.6)	**0.008^b^**
Atrial fibrillation	21 (0.2)	27 (0.2)	0.804^b^

^a^=t-test, ^b^=χ^2^, ^*^AChEI: Acetylcholinesterase inhibitors include donepezil, galantamine and rivastigmine.

### Medication use for people who lived alone with dementia

The binomial regression model (Model 1, Table 2), when controlled for age and gender, showed that people living alone with dementia were associated with increased odds of receiving donepezil (OR 1.11, 95% CI 1.04–1.20, p= 0.004) and reduced odds of receiving antidepressants (OR 0.91, 95% CI 0.84–0.98, p= 0.015). After adjusting for age, gender, deprivation scores, area of living (urban or rural) and the marital status in model 2, people who lived alone were less likely to receive antidepressants (OR 0.91, 95% CI 0.85–0.99, p= 0.024).

**Table 2:** Binary regression models assessing associations for medication and living alone.

	Model 1*			Model 2**		
	**Odds ratio**	**95% CI**	**P-value**	**Odds ratio**	**95% CI**	**P-value**
**Dementia medication**						
Memantine	1.06	0.99; 1.14	0.123	1.06	0.93; 1.08	0.876
Donepezil	1.11	1.04; 1.20	**0.004**	1.07	0.99; 1.15	0.093
Galantamine	1.08	0.94; 1.25	0.280	1.00	0.86; 1.16	0.986
Rivastigmine	0.95	0.82; 1.11	0.524	0.95	0.82; 1.10	0.501
**Comorbidity medication**						
Hypercholesterolemia	0.94	0.84; 1.04	0.223	0.94	0.84; 1.05	0.260
Diabetes	0.88	0.74; 1.05	0.145	0.89	0.75; 1.05	0.172
Hypertension	0.92	0.80; 1.05	0.211	0.92	0.80; 1.06	0.258
Chronic anticoagulation	0.97	0.87; 1.09	0.598	0.97	0.86; 1.08	0.567
Anxiolytics	0.94	0.87; 1.01	0.096	0.94	0.87; 1.01	0.082
Antidepressants	0.91	0.84; 0.98	**0.015**	0.91	0.85; 0.99	**0.024**
Parkinson’s disease	1.02	0.84; 1.25	0.829	1.02	0.84; 1.25	0.820
Thyroid hormone problems	1.08	0.92; 1.27	0.365	1.04	0.88; 1.22	0.683
Hypnotics	0.97	0.90; 1.05	0.460	0.99	0.92; 1.06	0.735
Antipsychotics	0.98	0.91; 1.05	0.549	0.96	0.89; 1.04	0.293
Atrial fibrillation	1.20	0.68; 2.13	0.535	1.28	0.71; 2.28	0.412

*Model 1 adjusted for age and gender.

**Model 2 adjusted for age, gender, deprivation, area of living, marital status.

### Living with non-adult caregivers and other people with dementia

Analysis between people living alone and people living with a non-adult (17 years old or less; n=581) showed that the former group were significantly younger [75 and 76 years respectively; t(9,389)= -2.809, p= 0.005], were more frequently females [65% and 56%; χ^2^(1)= 153.516, p< 0.001], were less likely to live in rural areas [31% and 35%; χ^2^(1)= 4.284, p= 0.038], less likely to be married [40% and 50%; χ^2^(4)= 53.298, p< 0.001] and more likely to receive donepezil [31% and 25%; χ^2^(1)= 8.466, p= 0.004]. In comparison to people living with a non-adult, people living alone with dementia were less likely to receive medication for hypercholesterolemia [7% and 10%; χ^2^(1)= 5.760, p= 0.016] and hypertension [4% and 7%; χ^2^(1)= 11.161, p= 0.001], as well as less likely to receive anxiolytics [20% and 24%; χ^2^(1)= 5.527, p= 0.019], antidepressants [16% and 19%; χ^2^(1)= 5.764, p= 0.016] and antipsychotics [20% and 26%; χ^2^(1)= 12.924, p< 0.001].

Finally, analysis was carried out between people living alone and people living with another person with dementia (n=278). People living alone were younger [75 and 78 years; t(9,091)= -4,483, p< 0.001], more frequently females [65% and 59%, χ^2^(1)= 3.881, p= 0.049], married [40% and 53%; χ^2^(4)= 25.738, p< 0.001], lived in rural areas [31% and 26%; χ^2^(1)= 3.878, p= 0.049], and lived in more deprived areas [mean= 5.42 and 5.84; t(8,869)= -2.371, p= 0.018] compared to people living with another person with dementia. People living alone were less likely to receive memantine [29% and 38%; χ^2^(1)= 10.072, p= 0.002], but more likely to receive medication for hypercholesterolemia [7% and 4%; χ^2^(1)= 4.164, p= 0.041].

## DISCUSSION

This study explored whether people living alone with dementia had differences with respect to their demographic parameters and comorbidity medications in comparison to people with dementia who lived with at least one other person. Almost 35% of people with dementia in Northern Ireland live alone in their own home. This rate is similar to the one-third of people living alone with dementia in the whole United Kingdom and in the United States [1], but lower to the 51% rate in Germany [1] and 46% in Sweden [2].

Similar to past research, our results showed that people living alone with dementia were more likely to be female, were less likely to be married and more likely to be widowed [1, 2, 4]. Moreover, a study in France showed that people living alone with dementia are more often widowed females because they live longer than males [4].

In contrast to research evidence from Sweden, Germany and France [1, 2, 4, 6], people living alone in our study were younger (mean= 75 years old) compared to people who lived with another person (mean= 77 years old). In Sweden, people who lived alone were older (81 years old) compared to people who lived with others (77 years old) [2], and living alone was associated with increased odds of receiving antidepressants, antipsychotics, and hypnotics and sedatives. The younger age of people who lived alone in our study may explain why living alone was not associated with the use of comorbidity medication and psychotropic drugs, and was associated with less odds of receiving antidepressants (Table 2). In past research people who live alone with dementia were more likely to receive psychotropic drugs, such as antidepressants or antipsychotics, possibly because living alone can be accompanied with feeling of loneliness and more severe behavioural problems [2, 6]. However, people living alone in past research were significantly older compared to people who lived with a caregiver and thus, the younger age of people living alone in our study can better explain the decreased use of psychotropic drugs in this group because they are less likely to have depressive symptoms and severe behavioural problems.

Furthermore, the younger age of people who lived alone may also explain why they received medication for less comorbidities compared to people who lived with a caregiver. Another possible explanation is that the presence of a caregiver is associated with better treatment adherence and management of any side effects [2] and thus, clinicians might be more likely to prescribe comorbidity medication to people who live with a caregiver and less likely to people who live alone.

People with dementia who lived alone were more likely to receive Donepezil; this may be because of their younger age and fewer comorbidities hence they may be able to tolerate it better [10]. Past research has shown that AChEIs are prescribed to younger PwD with better cognitive performance, while advanced age is associated with reduced likelihood of receiving AChEIs possibly because of a greater cognitive decline [11]. AChEIs are prescribed to people with mild to moderate dementia and memantine is more often prescribed to severe dementia. PwD often receive AChEIs alone in the first years following a dementia diagnosis and then they are often prescribed a combination of AChEIs and memantine, while the use of memantine alone is more frequent in more severe stages of dementia [11]. Some evidence has shown that the combination of an AChEIs and memantine has more benefits for PwD instead of prescribing them alone, and are safe and well tolerated to use [11–13]. This can explain our finding for the increased rates of receiving a combination of AChEIs and memantine in people who live with a caregiver because this group was significantly older than people living alone.

Although the results from this study contribute to important findings about drug treatment in dementia, several limitations should be considered. People with dementia were identified through their dementia medication and thus, our sample potentially included mainly people with Alzheimer’s disease, mixed dementia and Lewy Body dementia. In addition, people who lived alone could receive care from friends and relatives who did not live with them. Research has shown that people who live alone with dementia are often supported by their siblings, children and grandchildren [14]. The EPD only holds information on medication which was prescribed by a General Practitioner and dispensed by a pharmacist, therefore, at present, we have no way to account for medications which are prescribed over the counter and as a result we may have underestimated the comorbidity medications. Finally, the marital status was recorded only for people who were admitted to a hospital (65%) and this data was not available for the remaining 35% of people. Moreover, the marital status from the first hospitalisation was often not updated with data from later admissions.

To conclude, the present study showed that people living alone with dementia are equally or even less likely to be prescribed medication for comorbidities compared with people who lived with someone else. This can be explained by the absence of a caregiver as well as by the support provided by health and social care services in Northern Ireland. The present results should be considered by local authorities in health services for future planning. Finally, the public and voluntary sectors should continue to provide vital information for people who live alone with dementia, such as for staying safe and dealing with loneliness and depression [15].

## MATERIALS AND METHODS

### Population and demographic characteristics

Similar to previous studies, people with dementia (n= 25,418) were identified using data which was extracted from the Enhanced Prescribing Database (EPD). The first date that a dementia management medication was prescribed was used as a proxy for a dementia diagnosis [16, 9]. The EPD includes 80-90% of medication prescribed by General Practitioners and dispensed by a pharmacist in Northern Ireland. Data was extracted for people from January 2010 to December 2016. Data for whether people lived alone or whether more people were registered in this address, was extracted from the National Health Application and Infrastructure Services and reflected the living arrangements of people with dementia as of January 2010. This data also showed if a person with dementia lived with at least one adult, a non-adult (17 years old or less), or with another person with dementia. Demographic characteristics, such as gender, age, deprivation and the area of living were extracted from national datasets and are explained elsewhere [9]. Deprivation was identified using the Northern Ireland Multiple Deprivation Measure [17]. Higher scores for deprivation show less deprived areas. All data were linked through the Honest Broker Service following ethical approval from their committee prior to data access. People who had transitioned to a care home (n= 4,390) in January 2010 were excluded from the analysis.

### Dementia medication and comorbidities

Dementia and comorbidity medications were extracted through the EPD and are explained in our past research [9]. Comorbidities under investigation were hypercholesterolemia, diabetes, hypertension chronic anticoagulation, Parkinson’s disease and thyroid hormone problems. Data for medication was also extracted for anxiolytics, antidepressants, hypnotics and antipsychotics. Data was coded as 1 is a person received each of the medication, or 0 if not.

### Statistical analysis

Descriptive information is presented as frequency (n, %), means and standard deviation (SD). Comparisons between people who lived alone and people who lived with another person were conducted with chi-square tests for categorical variables, and with independent-samples t-tests for continuous variables. Chi-square and t-tests were also conducted to compare data between people living alone and people living with an adult, a non-adult or another person with dementia. A binary logistic regression model was used to estimate the odds ratios (ORs) and the 95% confidence intervals (CIs) for the relationship between living alone, demographic characteristics and medication use. A significance level of <0.05 was assumed for all statistical analyses. We analysed the data based on complete case analysis to deal with missing data.
